# DCBLD2 Affects the Development of Colorectal Cancer via EMT and Angiogenesis and Modulates 5-FU Drug Resistance

**DOI:** 10.3389/fcell.2021.669285

**Published:** 2021-05-19

**Authors:** Pan Xie, Fu-Qiang Yuan, Ma-Sha Huang, Wei Zhang, Hong-Hao Zhou, Xi Li, Zhao-Qian Liu

**Affiliations:** ^1^Hunan Key Laboratory of Pharmacogenetics, Department of Clinical Pharmacology, National Clinical Research Center for Geriatric Disorders, Xiangya Hospital, Central South University, Changsha, China; ^2^Institute of Clinical Pharmacology, Central South University, Changsha, China

**Keywords:** colorectal cancer, 5-FU, EMT, drug sensitivity, DCBLD2

## Abstract

**Background:** DCBLD2 is highly expressed in various cancers, including colorectal cancer. DCBLD2 overexpression promotes tumor occurrence, development, and metastasis. However, DCBLD2 sensitivity to chemotherapy drugs and its mechanism on tumor development are unknown.

**Methods:** DCBLD2 expression differences in cancer and normal tissues were obtained from GEO and TCGA databases. DCBLD2 influence on prognosis was also compared, and the database analysis results were verified via the analysis of clinical samples. GDSC database was used to analyze the effect of DCBLD2 expression difference on 5-FU drug sensitivity on tumor cells. CCK-8, clone formation, scratch, Transwell invasion and migration assays were used to assess DCBLD2 effects on the proliferation, metastasis, and 5-FU drug sensitivity on HCT116 and Caco-2 colorectal cancer cells. Angiogenesis and Matrigel plug assays were used to study the effect of DCBLD2 on angiogenesis. Q-RCR and Western Blot were used to analyze DCBLD2 impact on the EMT signaling pathway, and TAP-MS assay with Co-IP verification was used to identify the downstream target proteins binding to DCBLD2.

**Results:** Both database and clinical sample validation results showed that the expression of DCBLD2 in colorectal cancer tissues was significantly higher than that in normal tissues, leading to poor prognosis of patients. GDSC database analysis showed that DCBLD2 overexpression caused tumor cell resistance to 5-FU. The results of *in vitro* and *in vivo* experiments showed that the inhibition of DCBLD2 reduced the proliferation, migration and invasion of colorectal cancer cells, inhibited the angiogenesis of endothelial cells, and enhanced the drug sensitivity to 5-FU. The results of q-RCR and Western Blot experiments showed that the inhibition of DCBLD2 can suppress the EMT signal. The results of TAP-MS assay showed that the proteins bound to DCBLD2 were enriched to the Focal adhesion pathway. The results of Co-IP assay show that DCBLD2 can combine with ITGB1, the key factor of Focal adhesion pathway.

**Conclusion:** DCBLD2 may affect the development of colorectal cancer by regulating cell proliferation and motility, and modulate 5-FU resistance. Down-regulation of DCBLD2 can inhibit EMT signal and angiogenesis. DCBLD2 can combine with ITGB1, the key signal factor of the Focal adhesion pathway.

## Background

Colorectal cancer (CRC) is a gastrointestinal malignancy with high morbidity and mortality (Siegel et al., 2020a,b). Currently, surgical resection is the common clinical treatment for colorectal cancer. For patients with advanced and metastatic colorectal cancer, drug adjuvant therapy is usually provided to improve surgical outcomes and prevent tumor recurrence (Roselló et al., 2018). 5-fluorouracil (5-FU), oxaliplatin, irinotecan, cetuximab, and bevacizumab have been approved for clinical treatment of colorectal cancer. While drug adjuvant chemotherapy inhibits tumor recurrence and metastasis, prolonging the survival of patients, its efficacy is substantially reduced due to drug resistance (Longley et al., 2006; Wei et al., 2019).

In colorectal cancer, 5-FU is the first line drug promoted clinically. 5-FU is an inhibitor of thymidylate synthetase, which is a derivative of uracil where hydrogen is replaced by fluorine at position 5. By inhibiting the activity of Thymidylate synthase, it eventually inhibits the synthesis of purines, which leads to the reduction of DNA replication and repair, and plays a role in inhibiting the growth of tumor cells. On the other hand, 5-FU can be converted into 5-fluorouracil nucleoside *in vivo*, which is mixed into RNA in the form of pseudo-metabolites to interfere with protein synthesis (Xie et al., 2020). Since patients are prone to develop congenital or acquired resistance to 5-FU, the effectiveness of treatment can be seriously adversely affected.

Epithelial-mesenchymal transformation (EMT) is a biological process in which epithelial cells are transformed into cells with a mesenchymal phenotype by specific procedures. In this process, epithelial cells lose the connection with the basement membrane and obtain high migration, invasion and anti-apoptotic interstitial phenotypes (Nisticò et al., 2012). Previous studies have shown that EMT is an essential process in tumor invasion and metastasis. Besides, recent studies have shown that EMT indicates cell drug resistance (Du and Shim, 2016; Shibue and Weinberg, 2017). For instance, EMT signaling strongly predicts drug sensitivity, both from clinical response to chemotherapy and gene profilometry in breast cancer and non-small cell lung cancer (Farmer et al., 2009; Byers et al., 2013). EMT is associated with 5-FU resistance in colorectal cancer. However, its specific mechanism of action is unknown (Sun et al., 2017; Cheng et al., 2020).

In this study, we tried to explore the potential targets of 5-FU resistance in colorectal cancer through bioinformatics strategies and existing database resources. GDSC database was used to analyze the differentially expressed genes related to the IC50 value of solid tumor cells to 5-FU. GDC database and GEO database were used to analyze the TCGA colon cancer data treated with 5-FU and GSE17536 data set, and finally two groups of differentially expressed genes related to patient survival were obtained. Sixteen genes were obtained from the intersection of the above three groups of genes. The high expression of these genes may be associated with poor prognosis and 5-FU resistance in patients with colorectal cancer. Considering the significance of the expression difference and the innovation of the research, we decided to take the DCBLD2 gene as our follow-up research object.

The DCBLD2 gene is a protein-coding gene located on chromosome 3. The encoded protein is mainly located in the cell membrane and cytosol (Kobuke et al., 2001). The gene was up-regulated at the mRNA level in highly metastatic lung cancer cell lines, and may be involved in angiogenesis, as well as tumor genesis and progression (Koshikawa et al., 2002; Nagai et al., 2007). The structure of the DCBLD domain is very similar to neuronectin. As a transmembrane protein, it has two Cub and Discoidin domains and acts as a common receptor for Semaphorin 3 and growth factors in axon guidance and angiogenesis (Geretti et al., 2008). DCBLD2 is highly expressed in various cancers, including colorectal cancer. DCBLD2 overexpression promotes tumor occurrence, development, and metastasis (Koshikawa et al., 2002; Feng et al., 2014; He et al., 2020). However, DCBLD2 sensitivity to chemotherapy drugs and its mechanism in tumor development and metastasis are unknown. In this study, we elucidate the effect and mechanism of DCBLD2 on the degree of malignancy and 5-FU treatment sensitivity of colorectal cancer through *in vivo* and *in vitro* studies.

## Materials and Methods

### Biological Information Analysis

The gene expression data of solid tumor cells and 5-FU treatment information were obtained from the GDSC^1^ database. Combining the above two sets of data to analyze the correlation between gene expression and IC50 value, the genes with positive correlation between expression value and IC50 were screened out. The differentially expressed genes were compared by “limma” package of R (fold change > 2, *P* < 0.05). RNA-seq data of TCGA colon cancer patients receiving 5-FU treatment in GDC^2^ database, RNA-seq data of CRC patients in GEO^3^ database, and the matched clinical data were downloaded. Combined with the overall survival time and gene expression value, the genes affecting the prognosis of patients were analyzed (*P* < 0.05). The reasons why we chose GSE17536 as our research object are as follows: (1) the sample size of the data set is large enough; (2) the patients in this data set have complete demographic, histologic grade, stage, and differentiation information; (3) the patients in this data set have information about receiving drug treatment.

### Clinical Specimens

The clinical study protocol was approved by the Ethics Committee of Xiangya Hospital (Changsha, China). All patients provided written informed consent, and a total of 141 patients participated in the study. Matched colorectal cancer tissues were collected from patients who underwent surgical resection at Xiangya Hospital between January 2017 and December 2019. The specimens were immediately frozen in liquid nitrogen or fixed with 10% formalin and embedded in paraffin. Immunohistochemical staining and scoring were performed on each slide. The expression status of DCBLD2 was determined by staining intensity and percentage of positive cells. The percentage of positive cells was divided into 0 (<10%), 1 (10–25%), 2 (26–50%), and 3 (>50%). Staining intensity was divided into zero staining (0), weak staining (1), moderate staining (2), and strong staining (3). The final score for each case was the product of the intensity score and the positive cell score.

### Cell Culture Conditions and Reagents

Human CRC cells lines (HCT116 and CACO-2) and Human Umbilical Vein Endothelial Cells (HUVECs) were purchased from the Chinese Academy of Sciences (Shanghai) and cultured in RPMI 1640 medium (Gibco, United States) containing 10% bovine fetal serum (Gibco, United States) in an incubator set to 37°C and 5% CO_2_.

### Construction of siRNA and shRNA

siRNA and shRNA targeting DCBLD2 were constructed using the following targets: sequence 1, 5’-CGATGTCAGTTTATTCCTA-3’; Sequence 2, 5’-GAACAGCAATGACCTCAAA-3’. For *in vitro* experiments, siRNAs were transfected into cells using iMAX (Invitrogen) for subsequent experiments in accordance with the requirements of the operating instructions. For *in vivo* experiments, Polybrene (5 μg/mL) was used to increase the infection efficiency of lentivirus on cells, and infection time of shRNA was 48 h. The cells with stable expression were screened with 4 μg/mL puromycin, and the expression of DCBLD2 gene was confirmed to be down-regulated by q-RCR.

### q-RCR (Quantitative Real-Time PCR)

Use Reverse Transcription Kit (Takara, Japan) to convert RNA to cDNA according to the instructions. Using SYBR Premix Dimer Eraser Kit (Takara, Japan) to configure the experimental system, q-PCR amplification was carried out on the LightCycler@480II/96 (Roche) instrument, and the primer sequence was attached to Supplementary Table 1. The internal control used in the qPCR experiment was GAPDH. The operation procedure of the instrument is as follows: pre-denaturation, 1 cycle, 95°C, 30S, 20°C/S. PCR amplification, 40 cycles, 95°C, 5S, 20°C/S; 55°C, 30S, 20°C/S; 72°C, 30S, 20°C/S. Dissolution curve analysis, 95°C 0S, 20°C/S; 65°C, 15S, 20°C/S; 95°C, 0S, 0.1°C/S. After getting the Ct value at the end of the program, Graph Pad Prism6 is used to analyze the experimental results.

### Western Blot

The total protein extracted from the cell was separated by SDS-PAGE and transferred to the PVDF membrane, and the latter was incubated in TBST solution containing 5% milk for 1 h to block the non-specific binding of the protein to the antibody. The membrane was incubated overnight in the specific antibody at 4°C, using β-actin as the internal control. After incubating 1 h with secondary antibody at room temperature the next day, it was developed on BIO-RAD CHEMIDOC XRS+ imaging system. The antibodies used in this experiment was attached to Supplementary Table 2.

### Cell Proliferation Assay

Twenty-four hours after siRNA transfection, the collected cells were seeded into 96-well plates with 1,000 cells per well. CCK-8 detection was performed after 12, 24, 48, 72, and 96 h, respectively. The absorbance of each well was measured at 450 nm using a microplate spectrophotometer. Taking time as the X axis and OD value as the Y axis, the growth curve of cells was plotted.

### Colony Formation Assay

Twenty-four hours after siRNA transfection, the collected cells were seeded into 6-well plates with 1,000 cells per well. Cells were put into an incubator for further culture for 12–14 days, and when single cells grew into single clones, they were fixed with 4% paraformaldehyde, stained with crystal violet, and photographed under a microscope. Three independent repeated experiments were carried out, and the average value was taken after counting the number of cell clones.

### Wound Healing Test

Cells were collected and inoculated in 6-well plates. When cells growth reached 95% confluence, the pipette nozzle was used to make scratches. The siRNA was transfected immediately and the cell migration was observed under a microscope at 0, 24, and 48 h after transfection.

### Cell Migration and Invasion Assay

Twenty-four hours after siRNA transfection, the cells were collected and counted. Invasion Assay requires 50 μl of 1:10 diluted Matrigel over the cells and 200 μl of cell suspension (containing 3 × 10^4^ cells) over the cells. After 24 h, the cells in the upper compartment that did not cross the membrane were removed, fixed with 4% paraformaldehyde, stained with crystal violet, counted under a microscope and photographed.

### Co-IP (Co-immunoprecipitation)

Collect cells (10^7^ cells, 10 cm petri dish) and add 1 ml precooled cell lysis buffer (The composition of the lysis buffer: 0.5M Hepes; 1.425M KCl; 0.05M MgCl_2_; 0.01M EDTA; 10% Triton-X100, diluted with ddH_2_O). The supernatant was immediately transferred to a new centrifuge tube after centrifugation at 4°C and 14,000 rpm for 15 min. Protein A/G agarose beads were washed twice with PBS, and 50% Protein A/G agarose beads working solution was prepared with cell lysis buffer. Fifty percent Protein A/G agarose bead working solution was added to the total protein at the proportion of 1:10. Add 4 μg antibody to the total protein and slowly shake the agarose bead-antigen antibody complex overnight at 4°C. The agarose bead-antigen antibody complex was collected, supernatant was removed, and washed with pre-cooled PBS for 3 times. Sixty microliter 2X loading buffer was added, and the loading sample was boiled for 10 min for Western blot test.

### Nude Mouse Tumor Model

All animal procedures were performed with the approval of the Local Medical Experimental Animal Care Commission of the Xiangya Hospital. Mice were randomly grouped (5 mice per group). Four-week-old male nude BALB/C mice were subcutaneously injected with HCT116/shDCBLD2 or control cells (10^7^ cells per mouse for the tumorigenesis experiment; 5 × 10^6^ cells per mouse for the tumorigenesis experiment of 5-FU treatment). For the tumorigenesis experiment of 5-FU treatment, when tumor volumes reached approximately 0.2 cm^3^, mice were subcutaneously injected with control vehicle (DMSO) or 5-FU (20 mg/kg every week). Body weights, tumor length (L) and width (W) were measured every 4 days. Tumor volumes were calculated by the following formula: V = 0.5 × LW^2^. Sixteen days later, mice were sacrificed. Subcutaneous tumor grafts were excised and weighed. The tumors were removed and fixed with 10% buffered formalin for hematoxylin-eosin (HE) staining.

### TAP-MS (Tandem Affinity Purification-Mass Spectrum)

The target gene expression vector with double label of Flag-strep was constructed to package lentivirus-infected target cells (TCT116), and overexpressed cell lines were Constructed, and the expression effect was detected by Western Blot. After cell lysis, the total protein was extracted, purified and eluted by streptactin resin and anti-flag antibody. The eluents of the experimental group and the control group were hydrolyzed with trypsin to obtain the polypeptide mixture of the two groups. LC-MS/MS were performed, respectively, to obtain the qualitative information of the protein. The protein interacting with the target protein can be obtained by subtracting the protein in the control group from the results of the experimental group.

### *In vivo* Matrigel Plug Assay

All animal procedures were performed with the approval of the Local Medical Experimental Animal Care Commission of the Xiangya Hospital. A total of 1 × 10^6^ HUVEC cells (NC or shRNA1/2) were suspended in 500 μl of Matrigel. Then, the mixture was injected into the mice subcutaneously within the dorsal area with a 25-gauge needle. Seven days later, the Matrigel plugs were taken out. Then, the plugs were fixed in 4% paraformaldehyde, embedded in paraffin, and sectioned for hematoxylin and eosin staining and immunohistochemistry with anti-human CD31 antibody.

### Immunofluorescence (IF)

Cells were cultured on confocal dishes, fixed in methyl alcohol, permeabilized with 0.5% Triton X-100, blocked with 5% BSA (in TBST), and incubated with anti-DCBLD2 or anti-ITGB1 (1:100) at 4°C overnight. Cells were then incubated with secondary antibody at room temperature for 30 min. Immunofluorescence (IF) images were obtained with confocal microscopy.

### Statistical Analysis

Data are presented as the mean ± SD for three separate experiments. One-way ANOVA and Bonferroni were employed for statistical analysis using GraphPad Prism 8 for windows software. *P* < 0.05 was considered to be statistically significant.

## Results

### Bioinformatics Prediction of DCBLD2 as a Potential Target of 5-FU Resistance in Colorectal Cancer

**FIGURE 1:**
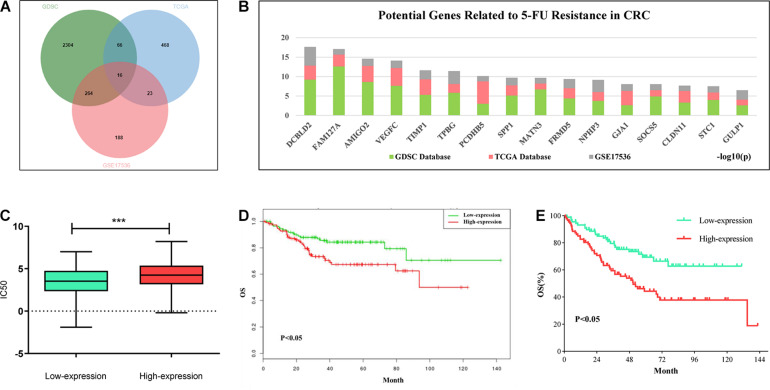
Comprehensive analysis of DCBLD2 mediated 5-FU resistance in CRC. **(A)** Strategies (Venn diagram) of screening 5-FU resistance-related genes in GDSC, GDC and GEO databases. **(B)** Results of screening 5-FU resistance-related genes in GDSC, GDC and GEO databases. Ranking of 16 candidate genes based on the sum of the significance of expression differences in the three data sets. **(C)** The effect of DCBLD2 expression on IC50 value of solid tumor cells was analyzed by GDSC database (***p < 0.001). **(D,E)** The effect of differential expression of DCBLD2 on overall survival of patients (Kaplan-Meier analysis) was analyzed using TCGA colon cancer data treated with 5-FU **(D)** and GSE17536 dataset **(E)**.

In order to explore the potential targets of 5-FU resistance in colorectal cancer, we downloaded 5-FU treatment information for all solid tumor cell lines and the gene expression data of the corresponding cells in the GDSC (Version 1) database. Combining the above two sets of data to analyze the correlation between gene expression and IC50 value, the genes with positive correlation between expression value and IC50 were screened out (fold change > 2, *P* < 0.05). The TCGA colon cancer data that received 5-FU treatment was downloaded from the GDC database and the colorectal cancer patient data of the GSE17536 data set was downloaded from the GEO database. In these two sets of data, genes that are highly expressed and related to the shortened survival of patients were screened out (*p* < 0.05). Based on the intersection of the above three sets of data, 16 candidate genes were obtained. The high expression of these genes may be related to the poor prognosis of patients with colorectal cancer and 5-FU resistance (Figure 1A). Among the above 16 candidate genes, taking full account of the significance of gene differential expression and the innovation of the research, DCBLD2 was selected as the next research object (Figure 1B). The solid cancer cells were grouped into DCBLD2 high and low expression groups. The high expression group had a relatively higher IC50 value in the GDSC database, indicating that DCBLD2 overexpression promotes drug resistance (Figure 1C). Similarly, colorectal cancer patients were divided into the DCBLD2 high and low expression groups. Patients with high DCBLD2 expression had a shorter survival time and a poor prognosis in the TCGA colon cancer patients receiving 5-FU treatment and the GSE17536 data set (Figures 1D,E).

### DCBLD2 Overexpression Is Associated With Poor Prognosis in Colorectal Cancer

**FIGURE 2:**
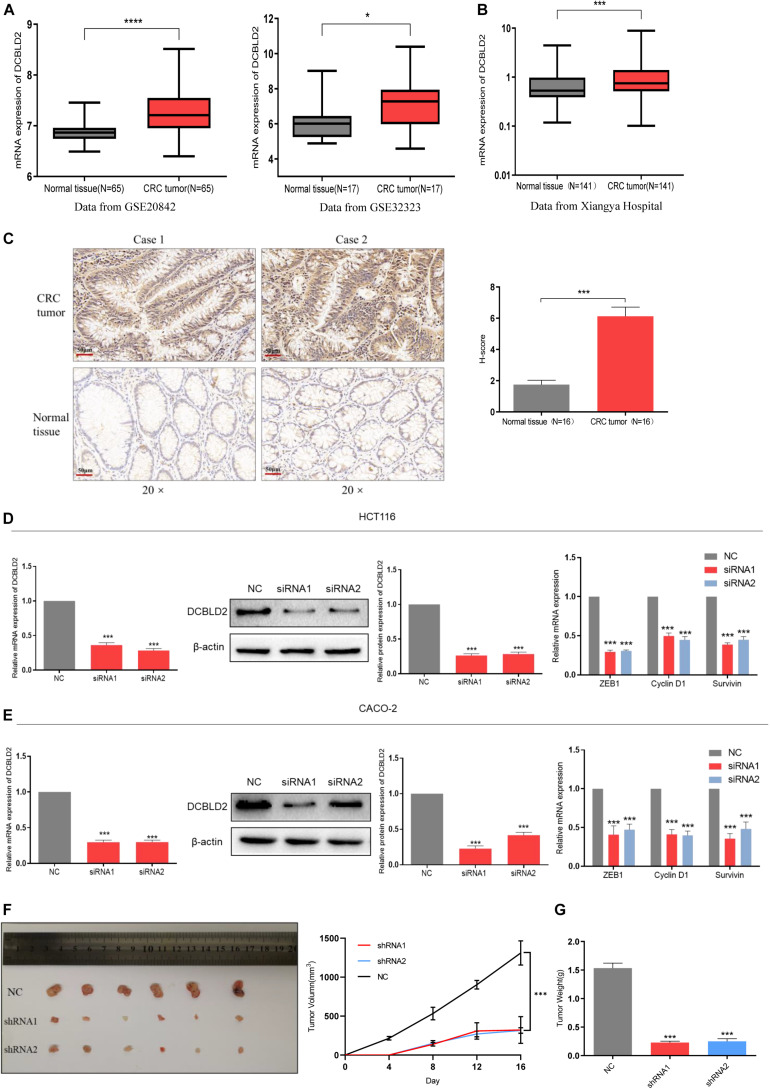
DCBLD2 expression is up-regulated and leads to poor prognosis in CRC. **(A)** The difference of DCBLD2 expression between cancer and normal tissues in the data sets GSE20842 (****p < 0.0001) and GSE32323 (*p < 0.05). **(B)** The difference of DCBLD2 mRNA level between cancer and normal tissues in the samples from Xiangya Hospital (*N* = 141, ***p < 0.001). **(C)** The difference of DCBLD2 protein level between cancer and normal tissues in the samples from Xiangya Hospital. The picture on the right shows the quantitative statistics of the differences in protein expression (*N* = 16, ***p < 0.001). **(D,E)** After transfection of siRNA in HCT116 **(D)** and CACO-2 **(E)**, the knockdown efficiency of DCBLD2 was tested by q-PCR experiment (***p < 0.001) and Western Blot experiment (***p < 0.001). The right figure shows the effect of down-regulation of DCBLD2 on the mRNA levels of tumor prognosis-related genes ZEB1, Cyclin D1 and Survivin (***p < 0.001; *n* = 3, Mann–Whitney test). **(F,G)** Tumor formation experiment in nude mice. Subcutaneous injection of HCT116/shDCBLD2 or HCT116/NC in 4-week-old male nude BALB/C mice (10^7^ cells per mice). Taking out the tumor and take pictures after 16 days. **(F)** Tumor volumes after DCBLD2 shRNA1/2 adenovirus treatment (***p < 0.001; 6 mice per group). tumor length (L) and width (W) were measured every 4 days. Tumor volumes were calculated by the following formula: V = 0.5 × LW^2^. **(G)** Tumor weights in mice after DCBLD2 shRNA1/2 adenovirus treatment (***p < 0.001; 6 mice per group).

The GSE20842 and GSE32323 datasets were obtained from the GEO database. DCBLD2 was significantly expressed in colorectal cancer tumor tissues compared with the normal tissues (Figure 2A). A total of 141 cases (colorectal cancer patient tumors and adjacent tissues) from Xiangya Hospital of Central South University were used. The gene expression was substantially higher in tumor tissues than in the adjacent tissues (Figure 2B). The well-preserved patient tissues (*N* = 16) were used for immunohistochemistry. Immunohistochemistry results were consistent with the qPCR results (Figure 2C). The above experimental results all show that the expression level of DCBLD2 in tumor tissues of colorectal cancer is higher than that in normal tissues. Then, we down-regulated the mRNA level of DCBLD2 gene in colorectal cancer cells HCT116 and CACO-2, and found that its mRNA and protein level was further down-regulated. In addition, the mRNA level of genes ZEB1, Cyclin D1 and Survivin related to tumor prognosis were also significantly down-regulated (Figures 2D,E). The results of *in vivo* experiments also showed that after down-regulating the expression of the DCBLD2 gene, the volume and weight of mouse tumors were suppressed (Figures 2F,G). Mouse tumor tissue immunohistochemical analysis showed that DCBLD2 down-regulation significantly suppresses protein level (Supplementary Figure 1).

### DCBLD2 Regulates CRC Cells and Mice Sensitivity to 5-FU

**FIGURE 3:**
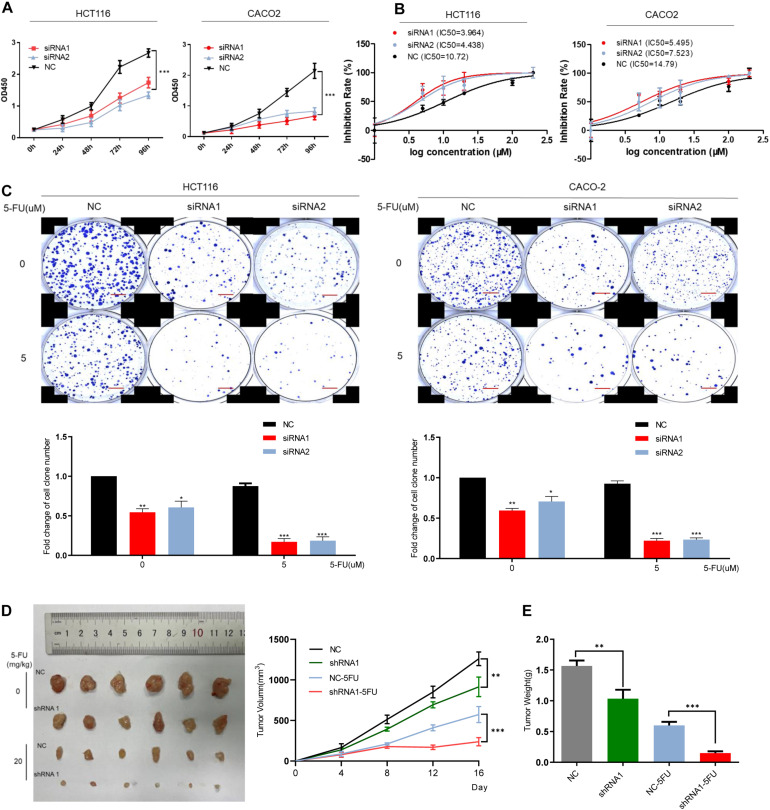
DCBLD2 regulates the sensitivity of CRC cells and mice to 5-FU. **(A)** The effect of DCBLD2 on CRC cell proliferation. After transfection of siRNA in HCT116 and CACO-2 cells, OD450 was measured every 24 h (***p < 0.001; *n* = 3, Mann–Whitney test). **(B)** Changes in the sensitivity of CRC cells to 5-FU treatment after down-regulation of DCBLD2. Statistical chart showed IC50 dose of 5-FU calculated from measurement of cell viability in different concentrations of 5-FU. **(C)** Representative images of colony formation of transfected CRC cells after 5-FU treatment. The figure below shows the quantitative statistics of the number of cell clones. Scale bars: 0.1 mm (*p < 0.05, **p < 0.01, ***p < 0.001; *n* = 3, Mann–Whitney test). **(D,E)** The effect of DCBLD2 on tumor formation ability and drug sensitivity of mice. Four-week-old male nude BALB/C mice were subcutaneously injected with HCT116/shDCBLD2 or control cells (5 × 10^6^ cells per mice). When tumor volumes reached approximately 0.2 cm^3^, mice were subcutaneously injected with control vehicle (DMSO) or 5-FU (20 mg/kg every week). Taking out the tumor and take pictures after 16 days. **(D)** Tumor volumes after DCBLD2 shRNA1/2 adenovirus and 5-FU treatment (**p < 0.01; ***p < 0.001; 5 mice per group). tumor length (L) and width (W) were measured every 4 days. Tumor volumes were calculated by the following formula: V = 0.5 × LW^2^. **(E)** Tumor weights in mice after DCBLD2 shRNA1/2 adenovirus and 5-FU treatment (**p < 0.01; ***p < 0.001; 5 mice per group).

The mRNA level of DCBLD2 was downregulated in colorectal cancer cells (HCT116 and CACO-2). The CCK-8 experiment showed that DCBLD2 down-regulation significantly inhibited cell proliferation (Figure 3A). The 5-FU gradient concentration treatment increased the cell drug sensitivity in the experimental group (Figure 3B). We conducted clone formation experiments under drug treatment and found that after the DCBLD2 was down-regulated, the number of cell clones was more suppressed by the drug (Figure 3C). The above phenotypic experiments proved that this gene may enhance the sensitivity of CRC cells to 5-FU by inhibiting proliferation. The results of *in vivo* experiments also showed that after down-regulating the expression of the DCBLD2, the volume and weight of mouse tumors were more suppressed by 5-FU (Figures 3D,E).

### DCBLD2 Regulates CRC Cell Sensitivity to 5-FU via Migration and Invasion

**FIGURE 4:**
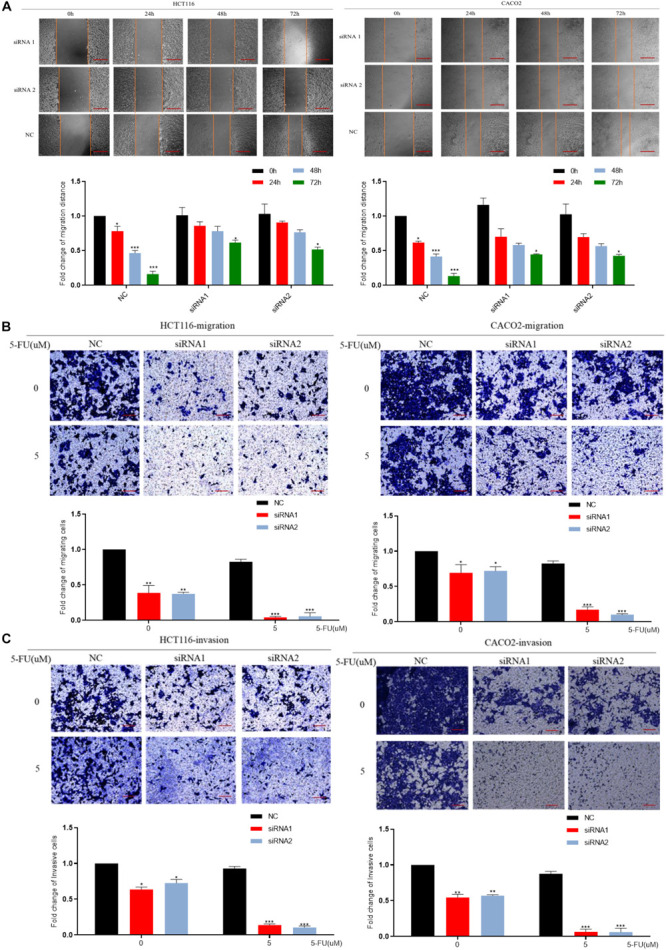
DCBLD2 regulates the sensitivity of CRC cells to 5-FU by affecting migration and invasion. **(A)** Scratch assay performed in HCT116 and CACO-2 cells transfected with control siRNA or DCBLD2 siRNA1/2 and treated with 5-FU or DMSO, respectively. Scale bars: 0.02 mm. **(B)** Transwell migration assay performed in HCT116 and CACO-2 cells transfected with control siRNA or DCBLD2 siRNA1/2 and treated with 5-FU or DMSO, respectively. Scale bars: 0.02 mm. **(C)** Transwell Matrigel invasion assay performed in HCT116 and CACO-2 cells transfected with control siRNA or DCBLD2 siRNA1/2 and treated with 5-FU or DMSO, respectively. Scale bars: 0.02 mm. All data are presented as the mean ± SD of three experiments (*p < 0.05, **p < 0.01, ***p < 0.001; *n* = 3, Mann–Whitney test).

We down-regulated the mRNA level of DCBLD2 in colorectal cancer cells (HCT116 and CACO-2). The result of the scratch experiment showed that the reduction of DCBLD2 expression would inhibit the migration ability of colorectal cancer cells (Figure 4A), similar to the Transwell migration experiment (Figure 4B). Besides, after giving cells 5 μM drug treatment, cell motility is more suppressed. Subsequently, we conducted Transwell Matrigel invasion experiment and found that this gene also affects the invasion ability of cells (Figure 4C).

### Down-Regulation of DCBLD2 Can Suppress Angiogenesis

**FIGURE 5:**
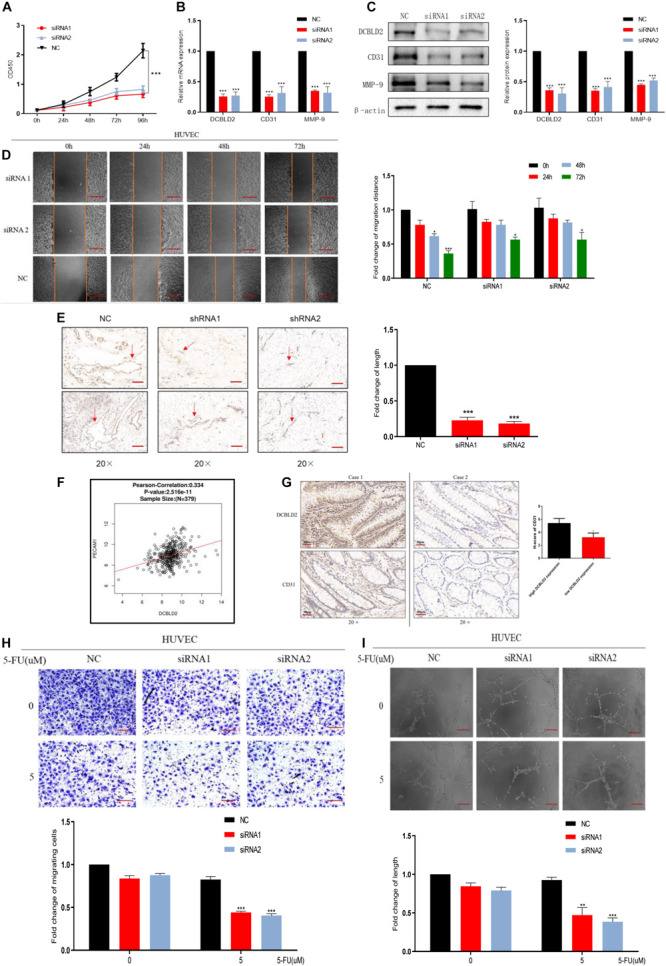
Down-regulation of DCBLD2 can suppress angiogenesis. **(A–D)** CCK-8 proliferation assays **(A)**, q-PCR assays **(B)**, Western Blot assays **(C)** and Scratch assay **(D)** performed in HUVEC cells transfected with DCBLD2 siRNA1/2. Scale bars: 0.1 mm (*p < 0.05, **p < 0.01, ***p < 0.001; *n* = 3, Mann–Whitney test). **(E)** Matrigel plug assay performed in mice, 7 days later, the Matrigel plugs was taken out from the mouse skin, and an immunohistochemical experiment was performed to analyze the angiogenesis ability. Arrows indicate the formation of microvessels in Matrigel. Scale bars: 0.02 mm (***p < 0.001; 5 mice per group). **(F)** Immunohistochemistry experiment to study the correlation between CD31 and DCBLD2 expression at the protein level among the tumor tissues of 16 CRC patients collected in Xiangya Hospital. Case 1 correspond to high DCBLD2 and case 2 to low DCBLD2 (*p < 0.05, *n* = 16). **(G)** Analyze the correlation between the expression of CD31 and DCBLD2 at the mRNA level in the TCGA data (*p* < 0.05, *n* = 379). **(H,I)** After 5-FU treatment (5 μM), perform Transwell Matrigel migration experiment and angiogenesis experiment in HUVEC cells transfected with control siRNA or DCBLD2 siRNA1/2. Scale bars: 0.02 mm (**p < 0.01, ***p < 0.001; *n* = 3, Mann–Whitney test).

The cBioPortal Database^4^ analysis showed that DCBLD2 significantly influences vascular development, indicating that DCBLD2 affects angiogenesis (Supplementary Figure 2A). DCBLD2 downregulation significantly inhibited cell proliferation and down-regulated angiogenesis-related protein levels, CD31 and MMP-9 in HUVEC cells (Figures 5A–C). The scratch test showed that DCBLD2 inhibition significantly inhibited HUVEC cell migration (Figure 5D). The results of Matrigel plug assay showed that the DCBLD2 will dramatically affect the angiogenesis ability. DCBLD2 down-regulation substantially decreased the vascular lumen size with incomplete structure (Figure 5E). Immunohistochemical experiments of 16 colorectal cancer patient tissues showed a significant positive correlation between the expression of DCBLD2 and CD31 at the protein level (Figure 5F). The TCGA data analysis showed that DCBLD2 expression was positively associated with CD31 expression at the mRNA level (Figure 5G). After the HUVEC cells were treated with 5-FU, the Transwell migration test, angiogenesis test and spheroidization test were performed. The results showed that after down-regulating the expression of DCBLD2 gene, the cell migration and tube formation capabilities were significantly more hampered by the drug (Figures 5H,I and Supplementary Figure 2B).

### Down-Regulation of DCBLD2 Can Suppress the EMT Signal

**FIGURE 6:**
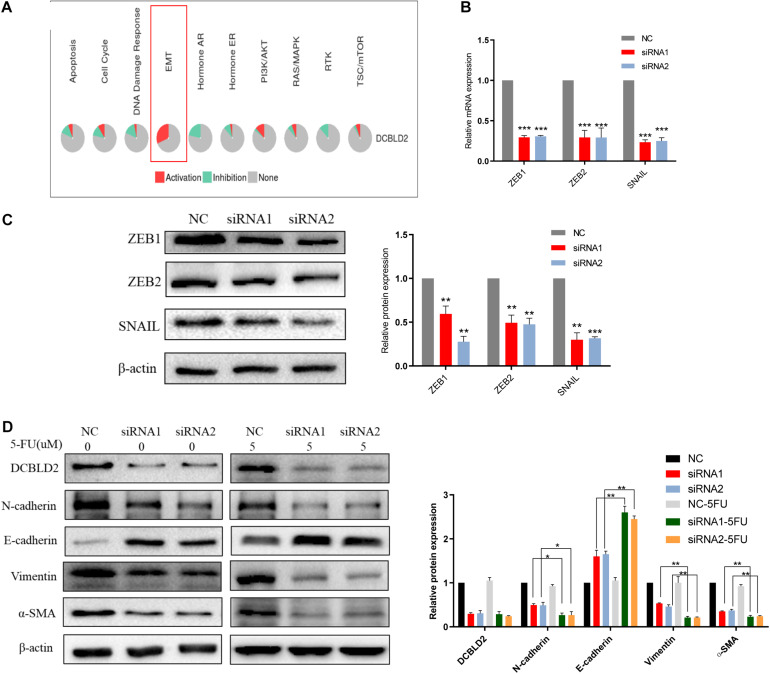
Down-regulation of DCBLD2 can suppress the EMT signal. **(A)** Bioinformatics prediction of the signal pathways affected by DCBLD2 in GSCALite Database. **(B,C)** The effect of DCBLD2 on the mRNA **(B)** and protein **(C)** level of the transcription factors ZEB1, ZEB2 and SNAIL upstream of EMT. Forty-eight hours after transfection of siRNA into HCT116 cells, RNA and protein were extracted for q-PCR and Western Blot experiments (***p < 0.001; *n* = 3, Mann–Whitney test). **(D)** Forty-eight hours after transfection of siRNA into HCT116 cells, changes in the protein level of N-cadherin, E-cadherin, Vimentin and α-SMA, the key protein factors of EMT after 5-FU or DMSO (5 μM) treatment detected by Western Blot experiment (*p < 0.05, **p < 0.01, ***p < 0.001; *n* = 3, Mann–Whitney test).

We conducted bioinformatics predictions on the signal pathways affected by the DCBLD2 in the GSCALite Database, and found that the most significant regulatory effect of this gene is the EMT signal (Figure 6A). DCBLD2 downregulation significantly suppressed mRNA and protein levels of the transcription factors, ZEB1, ZEB2, and SNAIL upstream of EMT in HCT116 cells (Figures 6B,C). We treated HCT116 with drugs and found that after DCBLD2 was down-regulated, the protein expression levels of EMT’s key protein factors N-cadherin, E-cadherin, Vimentin, and α-SMA changed more than the NC group (Figure 6D).

### DCBLD2 Can Combine With ITGB1, the Key Signal Factor of Focal Adhesion Pathway

**FIGURE 7:**
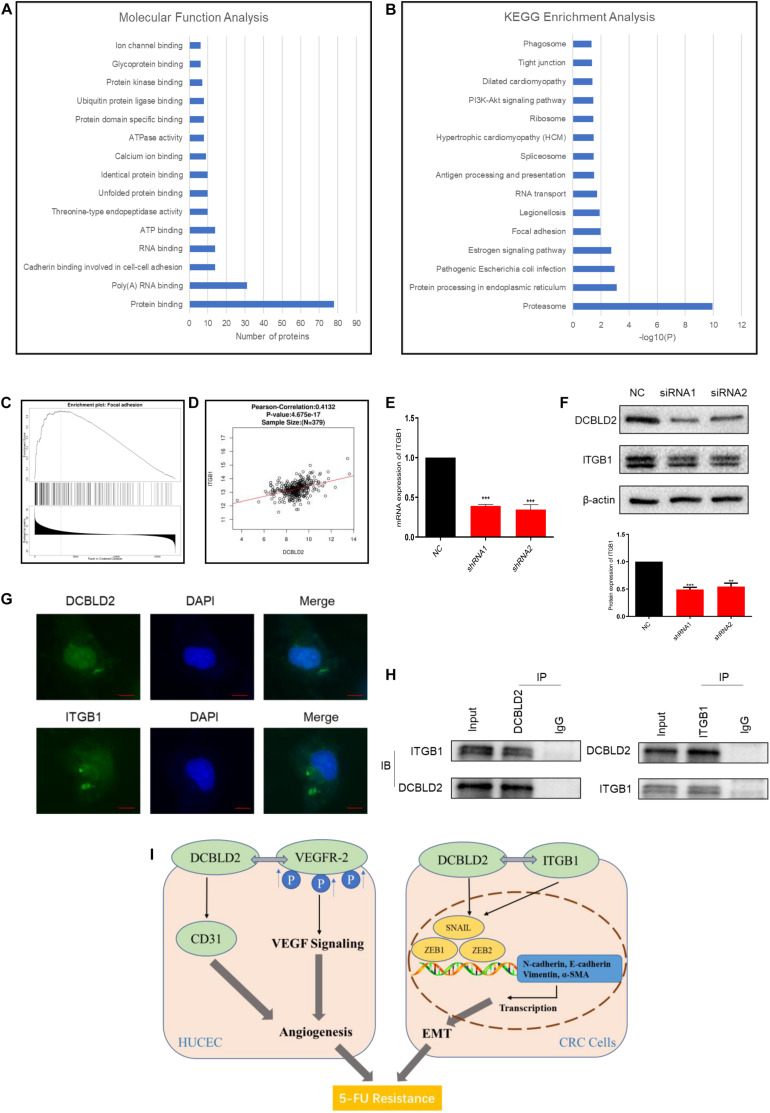
DCBLD2 can combine with ITGB1, the key signal factor of focal adhesion pathway. **(A,B)** Molecular Function Analysis **(A)** and KEGG Enrichment Analysis **(B)** of proteins screened out by TAP-MS experiment. **(C)** Enrichment analysis of DCBLD2 downstream genes obtained from analysis of TCGA data of patients with colorectal cancer. **(D)** Correlation between the expression of DCBLD2 and ITGB1 at the mRNA level obtained from analysis of TCGA data of patients with colorectal cancer (*p* < 0.05, *n* = 379). **(E,F)** Study the effect of DCBLD2 on the mRNA and protein expression levels of ITGB1 by q-PCR **(E)** and Western blot experiments **(F)** (***p < 0.001; *n* = 3, Mann–Whitney test). **(G)** Immunofluorescence assay to detect the localization of DCBLD2 and ITGB1 in HCT116. Scale bars: 5 μm. **(H)** Co-IP of DCBLD2 and ITGB1. HCT116 cells were immunoprecipitated using DCBLD2 or ITGB1 Abs and immunoblotted with ITGB1 or DCBLD2 Abs, respectively. **(I)** A graphical abstract of this study.

We performed a TAP-MS experiment on colorectal cancer cell (HCT-116). After two-step purification, 88 proteins that may bind to DCBLD2 protein were obtained. For the above proteins, we conducted Molecular Function Analysis and KEGG Enrichment Analysis, and found that these proteins mainly affect the binding of proteins in the cell (Figure 7A) and affect the focal adhesion pathway (Figure 7B). The TCGA data analysis showed that the downstream DCBLD2 gene was enriched in the focal adhesion pathway in colorectal cancer (Figure 7C), and DCBLD2 and the key signal factor ITGB1 of the focal adhesion pathway show a significant positive correlation in the mRNA expression level (Figure 7D). DCBLD2 downregulation significantly downregulated the mRNA and protein levels of ITGB1 in HCT116 (Figures 7E,F). DCBLD2 and ITGB1 showed significant polarity in the expression and location on the cell membrane, consistent with focal adhesion phenotype (Figure 7G). Finally, we further confirmed that DCBLD2 and ITGB1 can be combined with each other through Co-IP experiments (Figure 7H).

## Discussion

Colorectal cancer is a common gastrointestinal malignant tumor with high morbidity and mortality. DCBLD2, a protein-coding gene located on chromosome 3, is highly expressed in various tumors. Studies have shown that DCBLD2 overexpression promotes tumor invasion and migration, causing poor prognosis (Hofsli et al., 2008; Kim et al., 2008; Cheng et al., 2014; Fukumoto et al., 2014; Topkas et al., 2014). In this study, the database and clinical sample verification results showed that DCBLD2 expression substantially higher in colorectal cancer tissues than in normal tissues, causing a poor prognosis. CCK-8, clone formation, scratching, and Transwell experiments showed that the down-regulation of DCBLD2 inhibited the proliferation of CRC cells, and when the growth ability of cells is inhibited, the ability to migrate and invade is also inhibited. The angiogenesis and Matrigel plug experiments showed that DCBLD2 inhibition hinders angiogenesis, similar to *in vivo* experiments.

5-FU is the first-line drug for colorectal cancer (monotherapy or combination chemotherapy). However, congenital or acquired resistance significantly influences 5-FU efficacy. Therefore, further studies are needed to improve 5-FU efficacy. In this study, the solid cancer cells were grouped into DCBLD2 high and low expression groups. The high expression group had a relatively higher IC50 value in the GDSC database, indicating that high DCBLD2 expression has a lower drug sensitivity. Similarly, high DCBLD2 expression had a shorter survival time and a poor prognosis in the TCGA data and the GSE17536 data sets, suggesting that DCBLD2 is a potential 5-FU resistance target. Results of *in vivo* and *in vitro* experiments showed that DCBLD2 regulated the sensitivity to 5-FU via cell proliferation, migration and invasion.

With regard to the mechanism of the effect of DCBLD2 on the development of colorectal cancer, our study proposed two independent mechanisms. (1) DCBLD2 can promote angiogenesis. (2) DCBLD2 promotes the EMT process of colorectal cancer cells. From the perspective of tumor microenvironment, we believe that the two mechanisms of the role of DCBLD2 in the occurrence and development of colorectal cancer are complementary and superimposed (Figure 7I).

For the first mechanism, we proved that down-regulation of DCBLD2 cells can inhibit the motility and tube-forming ability of endothelial cells. In clinical samples, the expression of DCBLD2 and CD31 showed a significant positive correlation at both mRNA and protein levels. We down-regulated the expression of DCBLD2 gene in HUVEC cells, and the expression of CD31 decreased significantly at mRNA and protein levels. The results of Matrigel plug assay and angiogenesis experiment showed that with the silencing of DCBLD2, the cells lost their endothelial characteristics to some extent. In addition, previous studies have shown that DCBLD2 can bind with VEGFR-2 and inhibit the association between PTP1B, TCPTP, VE-cadherin and VEGFR-2, thus promoting the process of VEGFR-2 phosphorylation induced by VEGF, and finally up-regulating the VEGF signal (Nie et al., 2013). Our experimental results provide supplementary evidence at the phenotypic level for this study.

For the second mechanism, we first used TCGA data to analyze the relationship between the expression of DCBLD2 gene and the activity of 10 well-known tumor-related pathways in patients with colorectal cancer, and found that this gene has the greatest impact on EMT and can activate this pathway. We down-regulated the expression of DCBLD2 in colorectal cancer cells, and the mRNA and protein levels of transcription factors upstream of EMT signal, ZEB1, ZEB2 and SNAIL significantly decreased, which suppress epithelial genes and activate a mesenchymal expression program (Lin et al., 2013; Kotiyal and Bhattacharya, 2016). In the process of EMT, the polarity of epithelial cells is lost, the contact with surrounding cells and stromal cells is reduced, and the ability of cell migration and movement is enhanced. The decrease of E-cadherin level can lead to the decrease of cell adhesion and make cells easy to invade and metastasize, which is considered to be the most prominent feature of EMT. At the same time, the cells acquired interstitial phenotype, which was characterized by increased expression of Vimentin, N-cadherin and α-SMA (Thiery et al., 2009). We detected the expression changes of the above key proteins after down-regulation of DCBLD2 in colorectal cancer cells, and found that the expression of E-cadherin increased, while the expression levels of Vimentin, N-cadherin and α-SMA decreased, indicating that EMT was inhibited.

In order to study the specific molecular mechanism of the effect of DCBLD2 gene on EMT and angiogenesis, we searched for the downstream target protein that binds to DCBLD2 protein by TAP-MS experiment. The results of Molecular Function Analysis and KEGG Enrichment Analysis show that these downstream target proteins mainly affect the binding of proteins and affect the focal adhesion pathway. Focal adhesion is a type of adhesive contact between the cell and extracellular matrix through the interaction of the transmembrane proteins integrins with their extracellular ligands, and intracellular multiprotein assemblies connected to the actin cytoskeleton. Focal adhesions anchor the cell to the substratum and can mediate both mechanical and biochemical signaling (Zamir and Geiger, 2013; Kuo, 2014). Functionally, single cell polarity directly participates in early attachment and regulates intercellular focal adhesion, thus affecting EMT and angiogenesis (Bhattacharya, 2018; Lorentzen et al., 2018). We found six key proteins of focal adhesion pathway, SHC1, ACTB, ACTN1, COL1A1, FLNA, ITGB1, and MYL12B, in the results of mass spectrometry. Previous studies have shown that the activation of ITGB1/FAK pathway signal can promote EMT and angiogenesis in lung cancer, liver cancer and non-small cell lung cancer (Guo et al., 2020; Wang and Chang, 2020; Li et al., 2021). The expression and localization of DCBLD2 on the cell membrane showed obvious polarity like ITGB1 when HCT116 cells were cultured in 3D condition, which was consistent with the phenotype of focal adhesion. Finally, through the CO-IP experiment, we further confirmed that DCBLD2 and ITGB1 can combine with each other.

## Conclusion

In conclusion, we elucidate for the first time the potential role of DCBLD2 in 5-FU resistance in colorectal cancer. And our study found that DCBLD2 can promote EMT and angiogenesis, thus promoting the development of colorectal cancer. DCBLD2 binds with ITGB1, the key signal factor of the focal adhesion pathway, and focal adhesion is an important pathway known to regulate EMT. The results of this study may contribute to the development of biomarkers and treatment strategies for patients with CRC. However, there are still some shortcomings in our research. On the one hand, the effect of DCBLD2 gene on FAK signal cascade has not been analyzed in detail; on the other hand, how the combination of DCBLD2 and ITGB1 affects focal adhesion still needs to be further discussed. This is also an important direction of our future research, and we will systematically analyze and explore this topic from the perspective of tumor microenvironment.

## Data Availability Statement

The raw data supporting the conclusions of this article will be made available by the authors, without undue reservation.

## Ethics Statement

The studies involving human participants were reviewed and approved by the Ethics Committee of Xiangya Hospital. The patients/participants provided their written informed consent to participate in this study. The animal study was reviewed and approved by the Ethics Committee of Xiangya Hospital.

## Author Contributions

PX carried out most of the experimental work. F-QY and M-SH conducted the cell culture, stable cell line establishment, and *in vitro* functional assays. PX and XL conceived of the project, designed most of the experiments, and wrote the manuscript. WZ, H-HZ, and Z-QL supervised the project. All authors read and approved the final manuscript.

## Conflict of Interest

The authors declare that the research was conducted in the absence of any commercial or financial relationships that could be construed as a potential conflict of interest.

## Footnotes

1 https://www.cancerrxgene.org/

2 https://portal.gdc.cancer.gov/

3 https://www.ncbi.nlm.nih.gov/geo/

4 http://www.cbioportal.org/

## Abbreviations

CRC: colorectal cancer
5-FU: 5-fluorouracil
DCBLD2: discoidin, CUB and LCCL domain containing 2
EMT: epithelial-mesenchymal transformation
GDSC: genomics of drug sensitivity in cancer
TCGA: the cancer genome atlas
GEO: gene expression omnibus
qPCR: quantitative real-time PCR
Co-IP: co-immunoprecipitation
TAP-MS: tandem affinity purification-mass spectrum
IF: immunofluorescence.
