# Supplementary figures and images for: DCBLD2 Affects the Development of Colorectal Cancer via EMT and Angiogenesis and Modulates 5-FU Drug Resistance
Source: Front Cell Dev Biol. 2021 May 19;9:669285. doi: 10.3389/fcell.2021.669285 (PMC8170045; doi:10.3389/fcell.2021.669285)

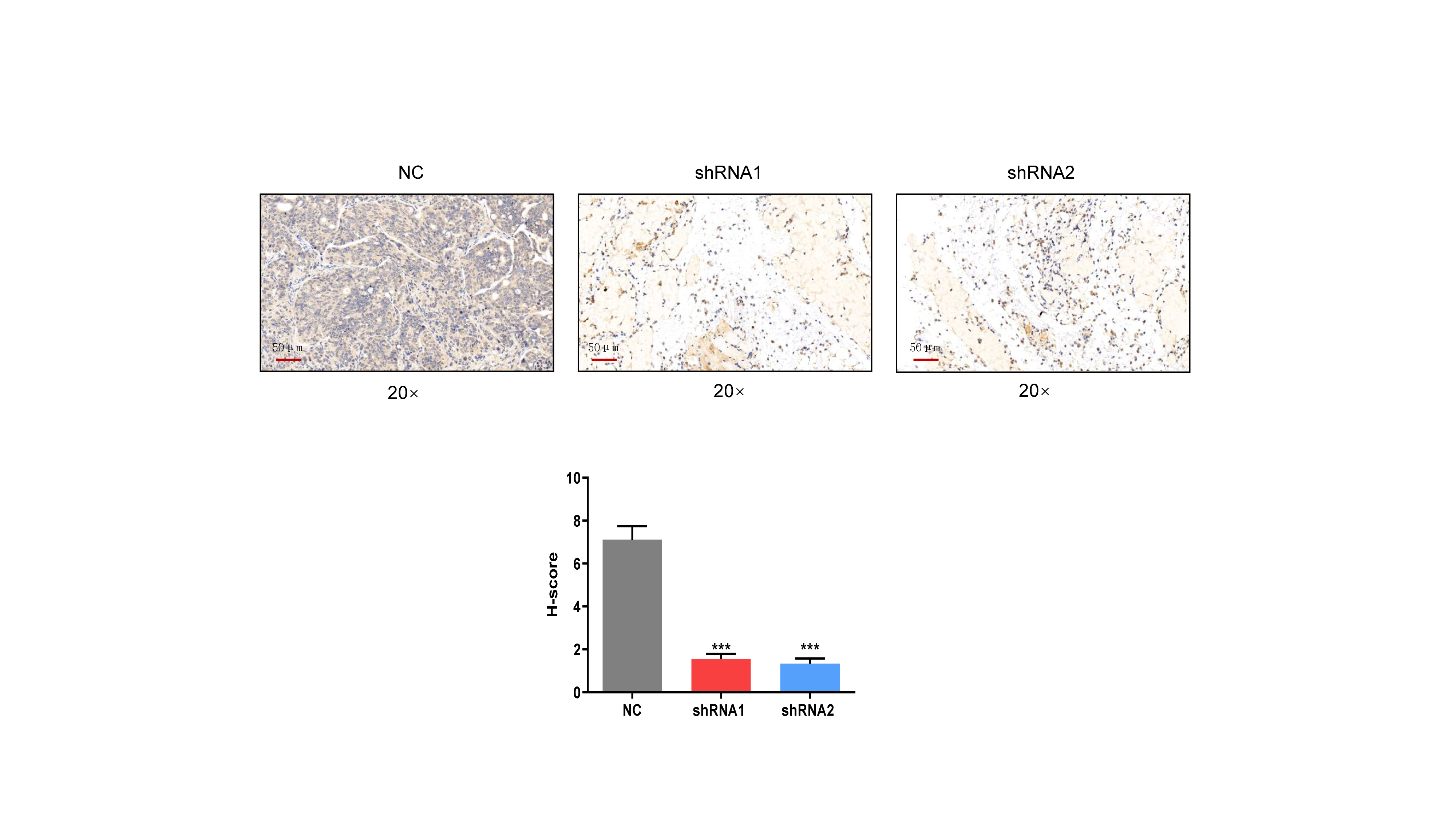

Supplement: Supplementary file 1 [file Image_1.TIF]

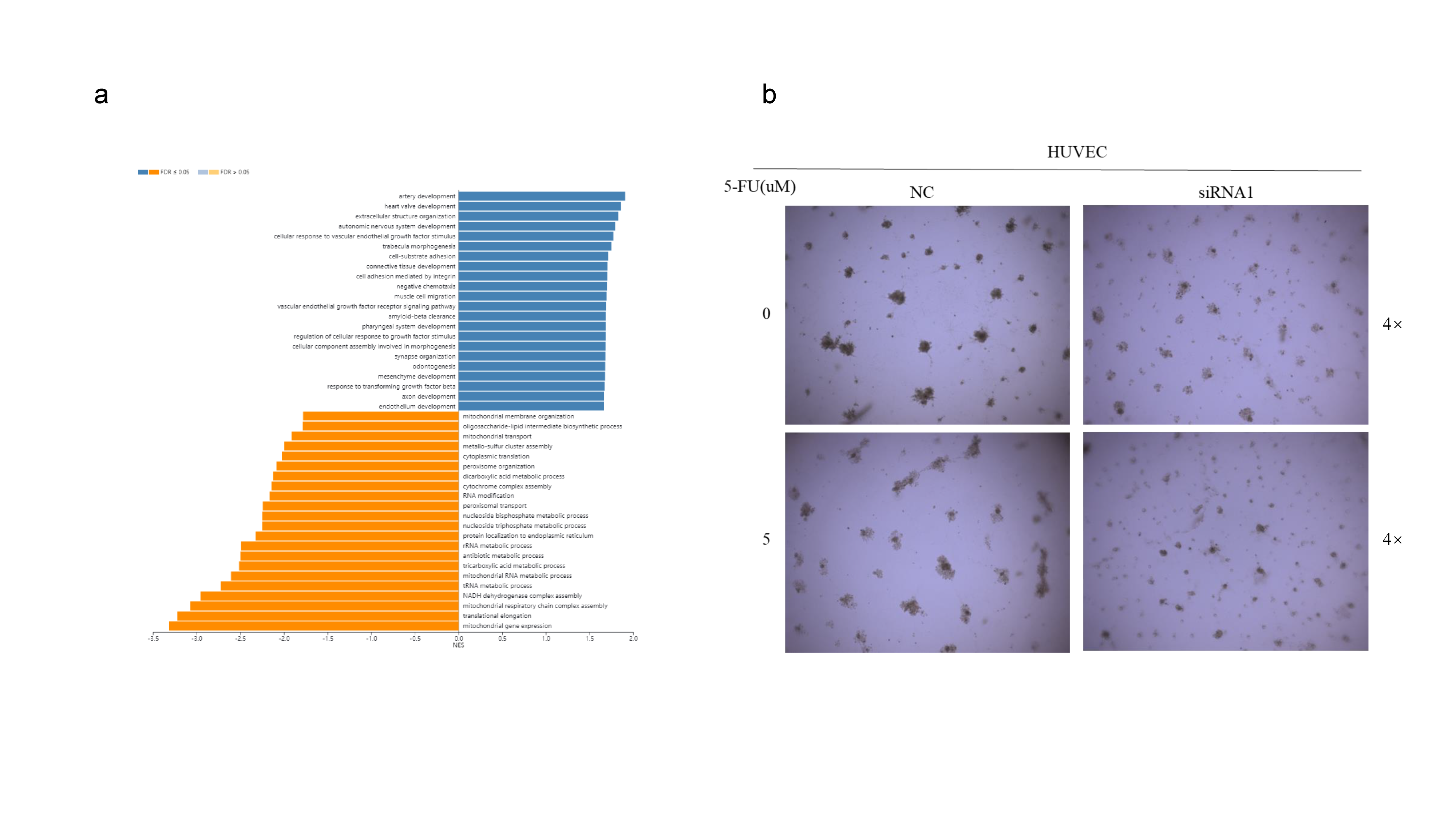

Supplement: Supplementary file 2 [file Image_2.TIF]
